# High-resolution X–ray diffraction datasets: Carbonates

**DOI:** 10.1016/j.dib.2022.108204

**Published:** 2022-04-22

**Authors:** Abduljamiu O. Amao, Bandar Al-Otaibi, Khalid Al-Ramadan

**Affiliations:** a Center for Integrative Petroleum Research, College of Petroleum Engineering and Geosciences, King Fahd University of Petroleum and Minerals (KFUPM), Dhahran 31261, Saudi Arabia; b Laboratory Support Services, College of Petroleum Engineering and Geosciences, King Fahd University of Petroleum and Minerals (KFUPM), Dhahran 31261, Saudi Arabia; c Geosciences Department, College of Petroleum Engineering and Geosciences, King Fahd University of Petroleum and Minerals (KFUPM), Dhahran 31261, Saudi Arabia

**Keywords:** X-ray diffraction, Carbonates, Spectra, Calcium carbonate rocks, Modelling

## Abstract

X–ray diffraction (XRD) analysis is a versatile and reliable method used in the identification of minerals in solid samples. It is one of the primary techniques geoscientists, mineralogist, solid-state chemists depend on to characterize the composition of unknown samples. In recent years there has been a growing interest among researchers to have readily accessible and large dataset to use to calibrate their experiment or to simply build various statistical models. Sadly, this is difficult to come by. Most well-curated datasets are propriety in nature and often too expensive for the average researcher. Additionally, when these datasets are available, they might not be suitable for purpose due to lack of proper coverage for certain a mineral of interest. For these reasons, we have carefully selected and curated samples rich in calcium carbonate that will be useful for various applications. Our dataset includes 1680 X-ray diffraction scans of samples collected from carbonate rich rock formations outcrops in Spain, Italy, and Saudi Arabia. They represent materials with total carbonate concentration range between 30-99%. The spectra were acquired on a Malvern PANalytical EMPYREAN Diffractometer system at two theta range 2- 70 and 0.01 step size. This dataset will be valuable to geoscientists, mineralogist, solid-state chemists, data scientists alike looking to design experiments, build mineralogical reference databases or statistical models with sufficient data points. We currently use the dataset in our own projects to develop comprehensive carbonate library and felt compelled to share.

## Specifications Table

**Table:** 

Subject	Earth and Planetary Sciences
Specific subject area	Data Mining, Spectroscopy and Statistical Analysis
Type of data	. xy files .raw files . xrdml file .csv figure table
How the data were acquired	The dataset was acquired on a Malvern PANalytical EMPYREAN Diffractometer system at two theta range 2- 70 and 0.01 step size, equipped with a Pixcel1D detector, a Reflection-transmission spinner (minimum step size Phi:0.1) sample stage, a Cu generator with K-Alpha1 [Å] =1.54060, K-Alpha2 [Å] =1.54443 and operated at a current of 40 mA and 45 kV volts. Custom scripts were written in R to transform, visualised, filtered, and processed and quantify the data with the help of packages such as “tidyverse”, “tidymodels”, “PowdR”. Conversion between XRD data types was accomplished in “PowDLL” software
Data format	raw analysed filtered
Description of data collection	Carbonate rock samples were first pulverised into powder, then loaded onto a Malvern PANalytical 27mm Sample Insert (9430 018 11271) with the aid of powder sample preparation kit (9430 017 70101) for making very flat pellet surface through back-loading. Subsequently, the pressed sample was supported by the bottom plate before loading to the system for analysis. The spectra were acquired on a Malvern PANalytical EMPYREAN Diffractometer system at two theta ranges of 2- 70 and 0.01 step size.
Data source location	King Fahd University of Petroleum and Minerals, Dhahran Saudi Arabia
Data accessibility	Repository name: Mendeley Data Data identification number: 10.17632/kxd48sck74.1 Direct URL to data: *https://data.mendeley.com/datasets/kxd48sck74/1*

## Value of the Data

• In recent years there has been a growing interest among researchers to have readily accessible and large dataset to use to calibrate their experiment or to simply build various statistical models.
• Sadly, this is difficult to come by most well-curated datasets are propriety in nature and often too expensive for the average researcher. Additionally, when datasets are available, they might not be suitable for purpose due to lack of proper coverage for a mineral of interest. For these reasons, we have carefully selected and curated sample rich in calcium carbonate that will be useful for various applications.
• Our dataset includes 1680 X-ray diffraction scans of samples collected from Spain, Italy, and Saudi Arabia.
• They represent materials with calcium carbonate concentration ranging between 30-99%.
• Geoscientists, mineralogist, solid-state chemists, and data scientists alike looking to design experiments or build statistical models with large data points.
• This dataset can be used to build databases, create data science model requiring large datapoints or be used as a reference dataset for comparing carbonates

## 1 Data Description

• Mendeley Data Repository (supplementary data): Folder one contains 1680 “. xy” carbonate spectra files in easily accessible format that can be open with a notepad.
• Mendeley Data Repository (supplementary data): Folder two contains 1680 carbonate spectra files in “. xrdml” PANalytical format.
• Mendeley Data Repository (supplementary data): Folder three contains 1680 carbonate spectra files in “. raw” Bruker/Siemens format.
• Supplementary data Table 1 attached: contains the major groupings of the 1680 carbonate spectra files based on their calcium carbonate composition.

**Table 1:** Summary statistic for the quantified spectra files including the mean, standard deviation (sd) the various quartiles (p0-p100) and the concentration range of each mineral depicted using histogram.

Mineral	Chemistry	Mean [%]	sd	p0	p25	p50	p75	p100	Histogram
*Calcite [%]*	CaCO_3_	69.48	24.92	5.24	55.36	79.39	88.39	99.57	
*Calcite_Magnesian [%]*	CaCO_3_	20.47	16.01	0.00	9.57	16.77	26.63	88.80	
*Vaterite [%]*	CaCO_3_	0.25	1.66	0.00	0.00	0.00	0.05	30.25	
*Smithsonite [%]*	ZnCO_3_	0.06	0.21	0.00	0.00	0.01	0.03	3.02	
*Siderite [%]*	FeCO_3_	0.07	0.08	0.00	0.01	0.05	0.09	0.79	
*Rhodochrosite [%]*	MnCO_3_	0.07	0.07	0.00	0.00	0.05	0.10	0.60	
*Dolomite [%]*	MgCO_3_	0.13	0.15	0.00	0.02	0.10	0.18	1.65	
*Monohydrocalcite [%]*	CaCO_3_ • H_2_O	0.13	0.15	0.00	0.02	0.10	0.18	1.65	
*Otavite [%]*	CdCO_3_	0.13	0.15	0.00	0.02	0.10	0.18	1.65	
Calcite Group Total [%]		**90.77**	**18.41**	**36.27**	**95.83**	**98.78**	**99.78**	**102.84**	
Other Minerals Total [%]		**9.32**	**18.43**	**0.00**	**0.29**	**1.21**	**4.27**	**64.12**
• Supplementary data table 2 attached: contains the quantified concentration calcite group of minerals of all the spectra files.
• Fig. 1 below is a sample spectrum from the dataset depicting a typical spectra file.

**Fig. 1:**
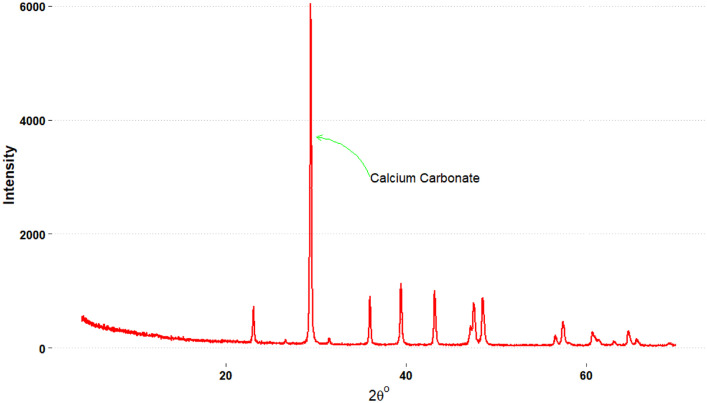
The scan example from a sample “Carbonates_1550” selected from the dataset
• Fig. 2 Result of Uniform Manifold Approximation and Projection (UMAP) and hierarchical clustering (HCPC) showing the variability among the carbonates spectra files.

**Fig. 2:**
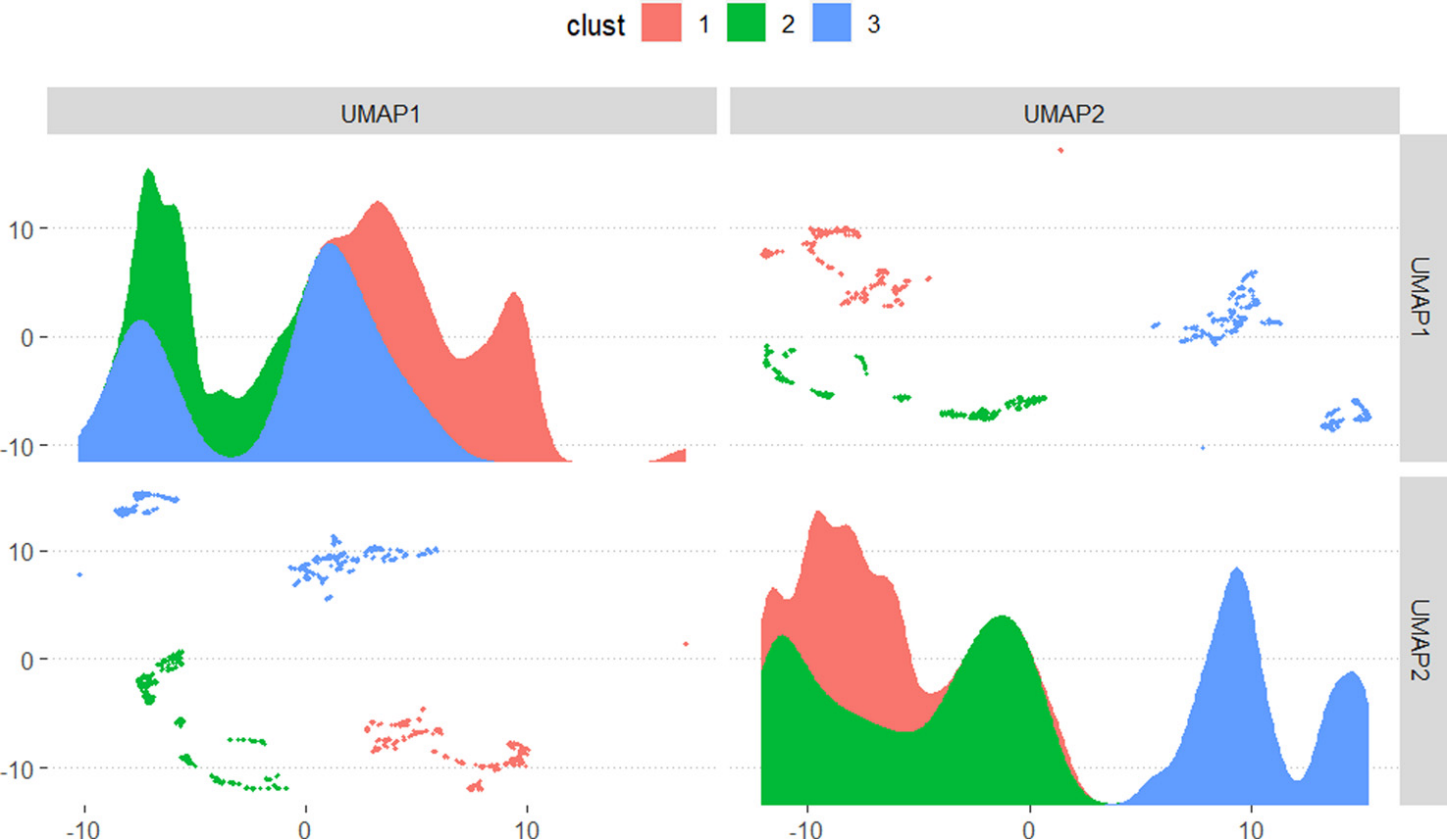
Visualising the differences among the shared files
• Fig. 3 A generalized pairs plot, offering a range of displays of paired combinations of categorical (Cluster) and quantitative variables (mineral Concentration). On the top, boxplots display the variability in concentration of the minerals. On the left, bar charts and scatterplots are used to display the main groups to which each of the spectra file belongs to based on cluster analysis while the scatterplots pair the concentration minerals. Pearson correlation is displayed on the right and range of variable distribution is available on the diagonal.

**Fig. 3:**
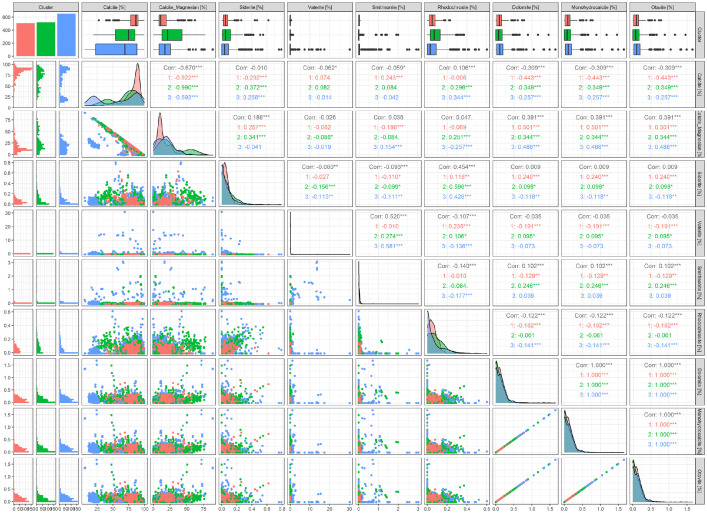
A generalized pairs plot, offering a range of displays of paired combinations of categorical (Cluster) and quantitative variables (mineral Concentration). On the top, boxplots display the variability in concentration of the minerals. On the left, bar charts and scatterplots are used to display the main groups to which each of the spectra file belongs to based on cluster analysis while the scatterplots pair the concentration minerals. Pearson correlation is displayed on the right and range of variable distribution is available on the diagonal.

## 2 Experimental Design, Materials and Methods

Samples for this dataset were collected from several research projects we've worked on in the past. The samples were collected from Spain, Italy, and Saudi Arabia representing over 10 carbonate formations. Spectra included in this dataset were carefully selected examining the percentage composition of calcium carbonate each individual file. The Calcite group of mineral is a loosely defined group dominated by Calcium carbonates. The group may also include other minerals such as dolomite, siderite, smithsonite, etc. (Table 1). Calcium carbonate (CaCO_3_) and its polymorphs (calcite, aragonite, and vaterite), are the focus of our dataset and someone of the most abundant and therefore important among biominerals. It is ubiquitous in nature and can be found in all rock types, hot springs, in caves, in ore deposits, and geyser deposits, etc., It also constitutes both structural and nonstructural components of living organisms and therefore its biomineralization makes a huge contribution to our ecological and geochemical systems [1], [2], [3]. A general non-linear dimension reduction technique i.e., Uniform Manifold Approximation and Projection (UMAP) was first applied to thousands of spectra files to sort out scans rich in carbonates and hierarchical clustering (HCPC) of UMAP embeddings was used to characterize the geochemical signatures and compare them to known carbonate files before they were quantified. If a spectrum was quantified and the calcium carbonate composition was below 30%, it was excluded from the list. Analyses were conducted using the R Statistical language (version 4.1.2; R Core Team, 2021) on Windows 10 × 64 (build 19044), using the packages hrbrthemes [4], powdR [5], tidymodels [6], purrr [7], report [8], datawizard [9], embed [10], rxylib [11], ggforce [12] and tidyverse [13]. Interconversion procedure between various formats of Powder X-Ray files was accomplished in PowDLL software [14]. The dataset was acquired on a Malvern PANalytical EMPYREAN Diffractometer system at two theta range 2- 70 and 0.01 step size, equipped with a Pixcel1D detector, a Reflection-transmission spinner (minimum step size Phi:0.1) sample stage, a Cu generator with K-Alpha1 [Å] =1.54060, K-Alpha2 [Å] =1.54443 and operated at a current of 40 mA and 45 kV volts. PowdR” an open-source software can be used to explore all the file types and to convert between the various XRD data types included in the dataset.

## Ethics Statements

 

## CRediT Author Statement

**Abduljamiu O. Amao:** Conceptualization, Methodology, Software, Formal analysis, Data Curation **Bandar Al-Otaibi**: Data curation, Investigation, **Khalid Al-Ramadan**: Funding acquisition, Writing - Review & Editing, Resources, Validation

## Declaration of Competing Interest

The authors declare that they have no known competing financial interests or personal relationships that could have appeared to influence the work reported in this paper.

## Data Availability

High-resolution X–ray diffraction datasets: Calcium Carbonates (Original data) (Mendeley Data)

## Appendix Supplementary materials

Image, application 1
